# Conflict-associated wounds and burns infected with GLASS pathogens in the Eastern Mediterranean Region: A systematic review

**DOI:** 10.1186/s12879-025-10569-3

**Published:** 2025-02-07

**Authors:** Amelia Wild, Clare Shortall, Omar Dewachi, Carine Naim, Alex Green, Sarah Hussain, Aula Abbara

**Affiliations:** 1 Department of Medicine, Imperial College, London, UK; 2 Médecins Sans Frontiers, Operational Centre Amsterdam, Amsterdam, the Netherlands; 3 Department of Anthropology, Rutgers University, New Brunswick, NJ USA; 4 Médecins Sans Frontières, Operational Centre Brussels, Middle-East Medical Unit, Beirut, Lebanon; 5 Children’s Hospital of Philadelphia, Philadelphia, PA USA; 6 Department of Infection, Imperial College London. St Mary’s Hospital, Praed Street, London, W2 1NY UK

**Keywords:** Antimicrobial resistance, Antimicrobial stewardship, Multi-drug resistant bacteria, Armed conflicts, Eastern Mediterranean Region, Conflict, War wounds, Burns, Civilians

## Abstract

**Background:**

While the relationship between conflict-associated injuries and antimicrobial resistance is increasingly being elucidated, data concerning civilian casualties is sparse. This systematic review assesses literature focused on Global Antimicrobial Resistance Surveillance System (GLASS) Priority Pathogens causing infections in civilian wounds and burns in conflict-affected countries within the World Health Organisation’s Eastern Mediterranean Region Office (EMRO).

**Methods:**

A systematic literature review was conducted following Preferred Reporting Items for Systematic Review and Meta-Analyses guidelines. Five databases and grey literature were searched, identifying studies published from January 2010 to June 2024. Search terms included “wounds”, “burns,” “antimicrobial resistance”, and the twelve countries of interest. Included studies reported resistance of GLASS pathogens. Two reviewers used Covidence to assess papers for inclusion. Data were extracted into a spreadsheet for analysis. Where quantitative data were available, medians, interquartile ranges and percentages were calculated by pathogen and country.

**Results:**

621 records were identified; 19 studies met inclusion criteria. Nine of the papers were from Iraq, three from Libya, three from Lebanon, one each from Yemen and Gaza; two reported on conflict affected refugees in Jordan. A total of 1,942 distinct microbiological isolates were reported, representing all four critical and high priority GLASS pathogen categories. Among the isolates, *Staphylococcus aureus* was the most prevalent (36.3%). Median resistances identified: Methicillin resistant *Staphylococcus aureus (n* = *680)*: 55.6% (IQR:49.65–90.3%); carbapenem resistant *Pseudomonas aeruginosa (n* = *372)*: 22.14% (7.43–52.22%); carbapenem resistant *Acinetobacter baumannii (n* = *366)*: 60.3% (32.1–85%); carbapenem resistant *Klebsiella pneumoniae (n* = *75):* 12.65% (9.73–34.25%)*;* ceftriaxone resistant *Escherichia coli (n* = *63)*: 76% (69–84.65%); ceftriaxone resistant *Klebsiella pneumoniae (n* = *40)*: 81.45% (76.73–86.18%). Only three studies had a low risk of bias.

**Discussion:**

Findings imply high rates of GLASS priority pathogens among wounded civilians in conflict-affected EMRO countries. However, evidence was heterogeneous, low quality and sparse in certain countries, highlighting the necessity of effective surveillance including standardised data collection. Improving primary data will facilitate the production of large, high-quality studies throughout the EMRO, including under-represented countries.

**Conclusion:**

Laboratory diagnostic capacity building and improved surveillance in conflict-affected settings in the Eastern Mediterranean Region are required to assess the burden of GLASS priority pathogens in vulnerable non-combatant populations.

**Supplementary Information:**

The online version contains supplementary material available at 10.1186/s12879-025-10569-3.

## Introduction

Antimicrobial resistance (AMR) is an escalating concern of global scope, with growing evidence linking conflict, forced displacement and AMR [1]. Civilians, especially those with conflict-related injuries, are particularly vulnerable due to several factors. These include the overuse or misuse of antibiotics without sufficient microbiological guidance, inadequate water, sanitation, and hygiene (WASH) in conflict-affected settings, overcrowding in displacement settings, and limited access to healthcare [2, 3]. The impact of conflict on health systems and the environment in affected countries further exacerbates the spread of AMR [4]. Contributing factors include the weaponization of healthcare, the loss of healthcare workers and infection specialists due to direct violence or displacement, the breakdown of vaccination programs and other public health measures, and the disruption of healthcare access [5, 6]. Importantly, the degradation of diagnostic capabilities leads to an increased reliance on empiric, broad-spectrum antimicrobial regimens which further facilitates the development and dissemination of AMR [7].

Since the Arab Spring in 2011, many countries in the World Health Organisation’s (WHO) Eastern Mediterranean Regional Office (EMRO) region have been affected by conflict, disaster and/or political instability [8–10]. Acute conflicts often arise against a backdrop of previous conflicts or existing stressors, with an increasing number of protracted conflicts, such as those in Syria and Yemen, straining health systems*.* The current escalation in Gaza (from October 2023) is likely to exacerbate AMR, in an area burdened by high resistance rate. For example, prior data from the *Médecins Sans Frontières* supported surgical reconstruction project in Al-Awda hospital already showed that 65% of *Staphylococcus aureus* isolates were methicillin resistant, 35% of *Pseudomonas aeruginosa* isolates were both carbapenem and ceftazidime resistant and 25% of *Enterobacterales* were carbapenem resistant [11]. In Syria, a country facing protracted conflict, the fragmentation and breakdown of the health system has resulted in limited access to healthcare and microbiology services. Although in-country data on AMR are limited, the burden is likely high [12]. For instance, Abbara et al. reported that among Syrian refugees in a surgical hospital in Jordan, 66% of Gram-negative bacteria were multidrug-resistant, and 36.7% of *Enterobacterales* were carbapenem-resistant [13].

According to WHO’s Global AMR and Use Surveillance System (GLASS) dataset, countries of the EMRO have the highest rates of AMR globally. In 2019, AMR was associated with over 400,000 deaths in EMRO countries [14]. This alarming figure is attributed to the overuse and misuse of antimicrobial agents, limited antimicrobial stewardship and a dearth of public health regulation and mechanisms of enforcement. As of 2022, 17 of the 22 EMRO countries have National Action Plans for tackling AMR, only four are actively implementing these plans, and just two governments support nationwide AMR awareness campaigns—none of which are in the ten conflict-affected countries [15]. Truppa et al.’s systematic review of GLASS pathogens in all EMRO countries, both conflict and non-conflict affected, found high levels of multidrug resistant organisms (MDROs) such as *Enterobacterales* and methicillin-resistant *Staphylococcus aureus* (MRSA) [16]. However, the work also highlighted significant heterogeneity and poor standardisation among the 132 included studies, with limited data on conflict-associated wounds or burns.

MDROs are organisms that exhibit resistance to one or more agents across three or more antimicrobial classes [17]. Relative to infections attributable to susceptible organisms, infections with MDROs impart extensively-documented, heightened risks of morbidity and mortality, inclusive of longer hospital stays, repeat surgeries, and treatment failure [18]. The WHO bacterial priority pathogens list, updated in 2024, defines two distinct categories: 1) a critical pathogen group, including carbapenem-resistant *Acinetobacter baumannii,* third-generation cephalosporin-resistant and carbapenem-resistant *Enterobacterales,* and 2) a high risk pathogen group, including carbapenem-resistant *Pseudomonas aeruginosa* and MRSA [19].

While MDROs can occur naturally, their proliferation is exacerbated by the breakdown of healthcare systems and the exodus of healthcare workers observed in conflict zones. This situation contributes to suboptimal clinical care, antimicrobial stewardship, and infection prevention and control practices [4]. These challenges may occur alongside reduced capacity for surgical or nursing care, including external fixation, debridement, and wound care.

Though dedicated literature exists on combatant injuries, current conflicts increasingly impact civilians [20]. Moreover, the healthcare and diagnostics available to military personnel, particularly international personnel, often differ significantly from those accessible to civilians, even when injuries occur in the same country [21, 22]. For local fighters e.g. military or non-state armed groups, this distinction may be less clear cut with mixing of civilians and fighters leading to community and nosocomial transmission of MDROs. This becomes more pertinent still in besieged areas where this mixing is intensified as seen in Ukraine, Syria or Gaza.

Such discrepancies imply that data from military casualties may not accurately represent civilian populations.

The rationale for this study is that it will identify the content of available literature and where gaps exist in the evidence base; this will allow recommendations on where the evidence base can be improved and where recommendations around diagnostics and the clinical care of patients can be improved.

The aim of this systematic review is to review the GLASS WHO priority pathogens that have been isolated and characterised from conflict-associated injuries in civilian patients in the Eastern Mediterranean.

## Methods

### Search strategy

A systematic literature search was conducted on 8th June 2024 (capturing data from January 2010 to 8th June 2024) in accordance with the Preferred Reporting Items for Systematic Reviews and Meta-Analyses (PRISMA) guidelines [23]. Medline, EMBASE, Global Health, the Index Medicus for the Eastern Mediterranean Region and Scopus databases were searched. The Journal BMC Conflict and Health and Médecins Sans Frontières and International Committee of the Red Cross websites were searched for any further relevant literature. Thereafter, citation searching was completed for reference lists of selected articles. Subject headings (MeSH terms), key words and synonyms such as “antibiotic resistance”, “conflict” and “wounds” were searched in combination with the relevant countries. The full search strategy can be found in *Additional file 1.1.*

The search results were imported into Covidence (2024) and deduplicated. Title and abstract screening was completed independently by two authors to select articles for full text screening. Ultimately, 65 studies underwent full text screening according to predefined inclusion and exclusion criteria *(*Table 1*).* Full texts were obtained through university library services, or by contacting authors directly. If texts were not accessible after 3 weeks of contacting the authors, the article was excluded.

**Table 1:** Eligibility criteria used for inclusion of records for this review

Criteria	Inclusion	Exclusion
Population	WHO EMRO populations which have been affected by conflict between January 2010-January 2024^2^ with addition of two countries which host a large number of conflict-refugees (Jordan, Turkey.) Studies reporting on civilians with wounds or burns currently residing in conflict-affected WHO EMRO countries	Studies conducted in WHO EMRO countries which have not been listed as fragile or conflict-affected from January 2010-January 2024.^2^ Studies considering only combatant populations Studies considering heterogenous populations, but where results between subgroups were not disaggregated
Condition	Wound or burn infections with selected GLASS WHO Priority Pathogens: Critical Group: carbapenem resistant *Acinetobacter baumannii;* third-generation cephalosporin-resistant or carbapenem-resistant *Enterobacterales. Note that Extended spectrum beta-lactamase (ESBL) resistant Enterobacterales and aminoglycoside resistance were reported where available* High Risk Group: carbapenem-resistant *Pseudomonas aeruginosa;* Methicillin-resistant *Staphylococcus aureus* (MRSA).^22^ Wounds and burns that are either explicitly conflict-associated, or are directly inferable from reported patient characteristics following full text review	Presumed infections without laboratory-confirmed microbiological isolates Infections attributed to organisms that are not classified as GLASS Priority Pathogens Infections in sites other than traumatic injuries attributable to conflict (e.g. surgical site infections after elective procedures) Infections in wounds that are pre-existent (eg diabetic ulcers), or in patients with pre-existent hospitalization for non-conflict-associated conditions
Outcomes/ Context	Prevalence or incidence of GLASS priority pathogens Proportion isolates meeting above criterion with susceptibility characterization meeting AMR GLASS pathogen characteristics	Susceptibility results of isolates from heterogeneous sources reported without sub-group analysis, and within which conflict-associated wounds do not represent the majority (i.e. < 50%)
Study Design	Academic literature: - primary research published in academic journals - quantitative and mixed methods studies Grey Literature: - reports - policy briefs - conference abstracts - guidance documents - registered but unpublished clinical trials	- Case Reports or small case series: less than 10 patients (overall, or specifically with regards to conflict-associated wounds) - Systematic Reviews - Commentaries - Unavailable full texts Other formats - Blog posts - Social media - Book Abstracts - Video/ audio reports
Time period	January 2010 to June 2024 Studies where data from between January 2010-June 2024 is disaggregated from data outside of this period	Studies published outside of January 2010—June 2024, or where the data reported is from outside of this period
Language	Publications in English	Texts with no available English translation

## Selection criteria

### Eligibility criteria

Population criterion consisted of civilians, studied in a country of interest. Civilians were defined as non-military personnel; studies where the nature of participants was not explicitly stated as being military, were considered civilian. These countries were those in the WHO EMRO that were also classified as fragile or conflict-affected according to the World Bank (2010–2024) [8]. To avoid missing essential data from relevant populations, other EMRO countries not on the list but which host a large number of conflict-refugees were included; these include Turkey (where 3.6 million Syrian refugees reside) and Jordan (where 630,00 registered Syrian refugees reside) [24].

### Exclusion criteria

Wounds or burns that were pre-existent or with a mechanism unattributable to conflict were excluded. Where the mechanism and/or conflict-association was not specified, methods and context were assessed, and studies were excluded if non-conflict related wounds or burns were inferred.

## Data Extraction

Data were extracted and collated into a spreadsheet in Microsoft Excel. Each study was given a study identification number. Data were collected under the following headings: article information, study characteristics, results, and conclusions.

To assess AMR prevalence, numbers of patients, microbiological assays, and positive isolates (total, and of selected GLASS pathogens) were extracted into a separate data sheet *(Additional file 2.1).* To assess the resistance patterns of the GLASS pathogens, the proportion of agent-specific antimicrobial resistance for each pathogen was identified and extracted. Antimicrobial agents were selected based on GLASS definitions of priority pathogens and clinical relevance for wounds and burns [25, 26]. These are summarised in *Additional file 1.2.* Median values and interquartile ranges (IQR) were calculated across studies for selected antimicrobials.

Where only the susceptible proportions of antimicrobial susceptibility testing were reported, the remainder were deemed resistant. Intermediate susceptibility values were included as non-susceptible in calculations. Studies in which isolates were declared resistant but a range of values, or no values, were quoted were not included in calculations. Regarding studies in which samples were taken from sites in addition to wounds, only the profiles of wound isolates have been extracted where sub-group analysis was reported. In studies where in the susceptibility testing results of multiple pathogens were reported, data was only extracted from selected GLASS priority pathogens pertinent to conflict-associated injuries. Where results were quoted for genera rather than named bacteria, resistances were not included in calculations for individual pathogens.

## Study quality assessment

Study quality and risk of bias was assessed using the Joanna Briggs Institute Critical Appraisal Tool [27]. This tool assesses 8 domains, including study participant selection, study sample, study design (including measurement of the exposure and outcomes), and data analysis; see *Additional file 1.3* for full details. For each domain, articles were classified and scored according to their fulfilment of the criteria using: yes (3 points), no (0 points) and unclear (1 point). Risk of bias was categorised according to scores: > 21 = low risk, 15–21 = moderate risk, < 15 = high risk.

## Results

### Study selection and characteristics

**Fig. 1:**
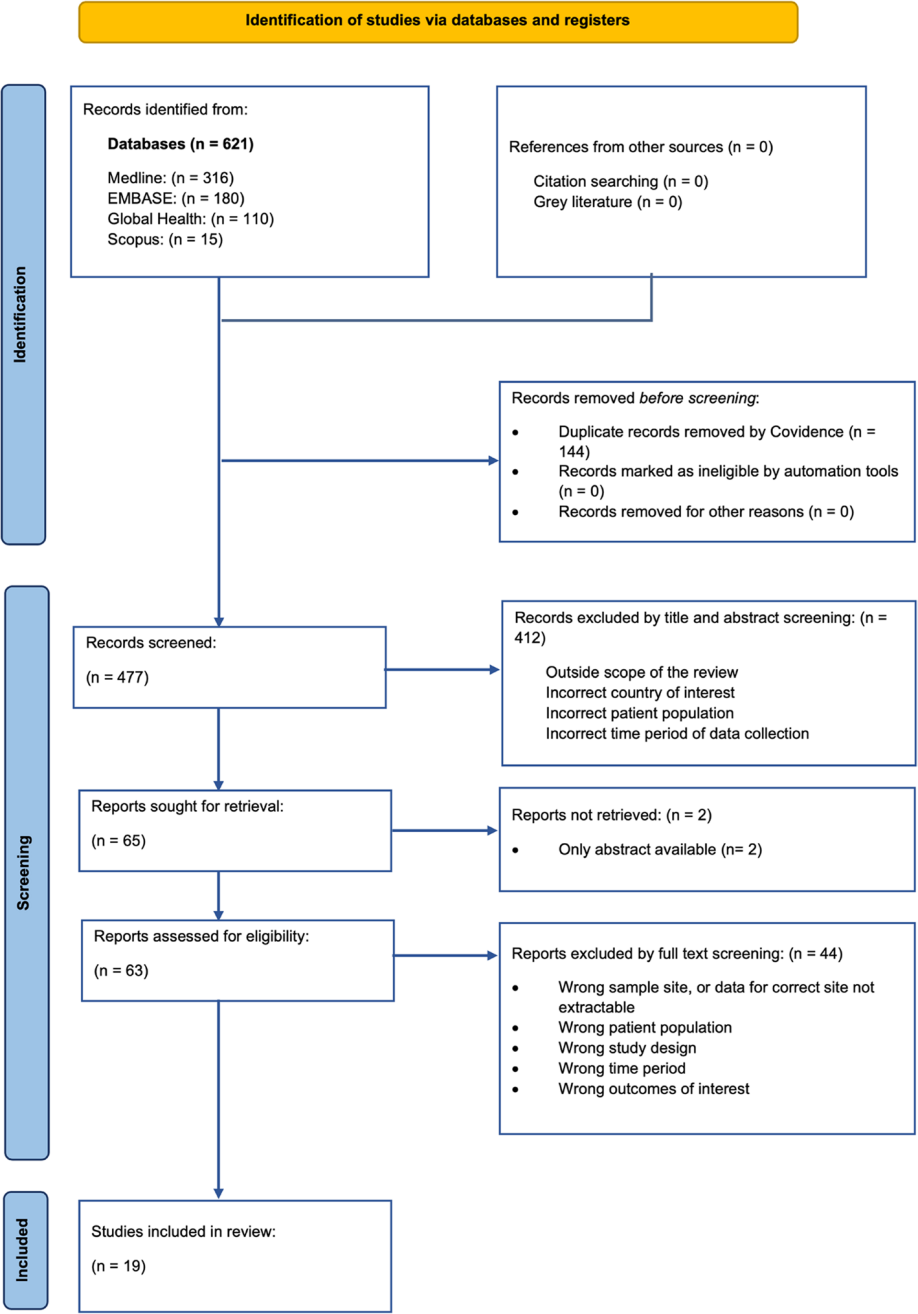
PRISMA flow diagram showing the study selection process for this systematic review

A total of 621 papers were identified from the search: 316 from Medline, 180 from Embase, 110 from Ovid Global Health, and 15 from Scopus. No studies were identified from the IMEMR database, grey literature, or citation screening. After removing 144 duplicates, title and abstracts of 477 papers were screened; full text screening for 65 papers was then conducted. 19 papers met the inclusion criteria and were included for analysis. Figure 1 outlines the selection process.

**Fig. 2:**
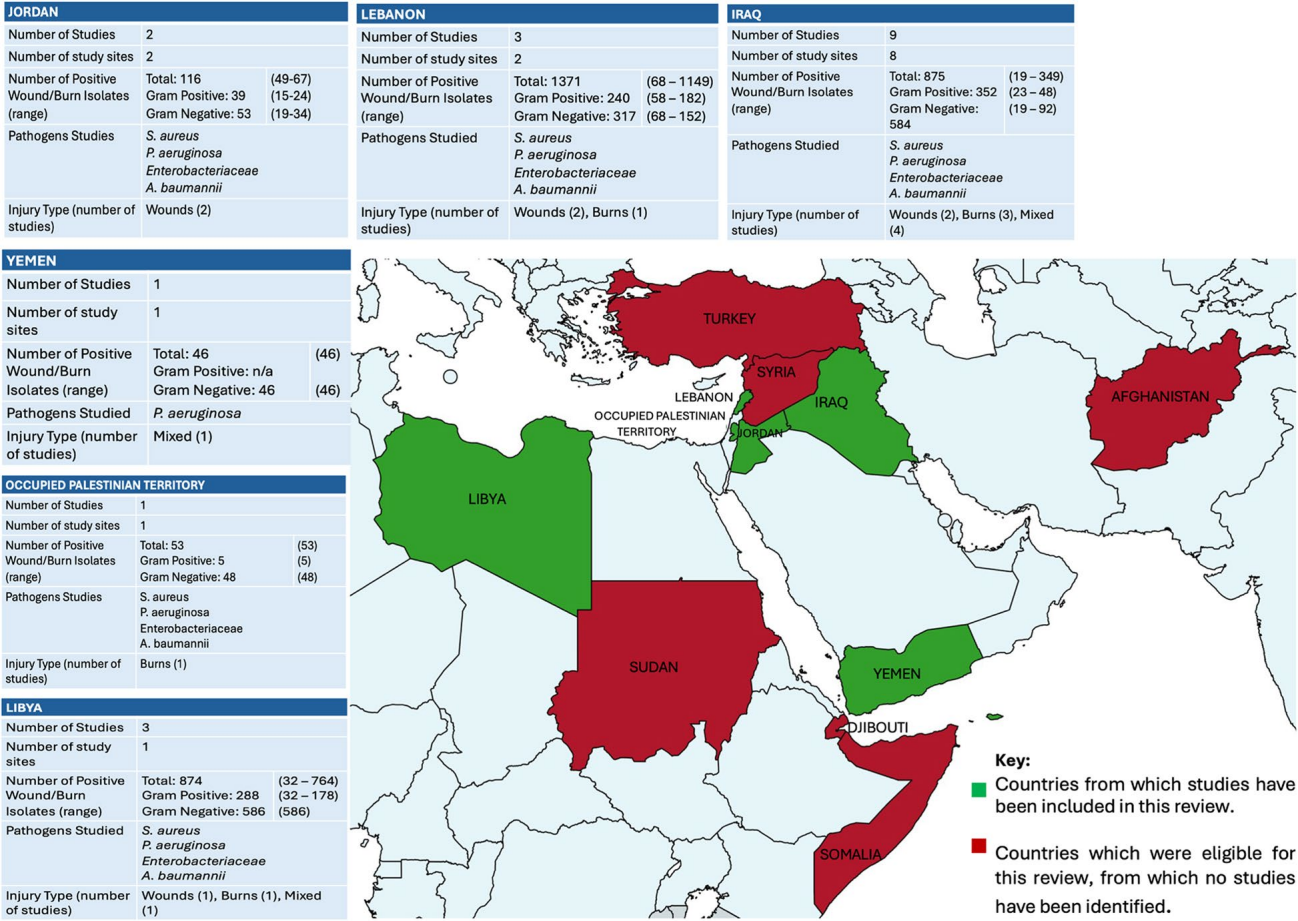
Map of the Eastern Mediterranean Regional Office. Map of countries eligible for inclusion in this review, colour coded according to their representation in this review. Figure includes characteristics of studies included in this review, collated according to country of publication

Nine of the included papers were from Iraq, three from Libya, three from Lebanon and one from each Yemen and Gaza; three reported on conflict-refugees in Jordan. Figure 2 shows study characteristics according to country. All studies were research articles. Two articles were excluded on full text screening as patients were pre-hospitalised and not deemed to represent conflict-related injuries [28, 29].

The 19 included studies were published between April 2013-January 2024, although 65% were published from 2018–2024. One study included results from pre-2010, which were disregarded [30]. All studies were conducted in hospital settings, including five in specialised burns units [30–34]. Where specified, mechanism of injuries varied, including gunshots, shrapnel injury, blunt trauma, burns, and orthopaedic complications [32, 35–39]. Six of the included studies reported data from burn wounds exclusively [30, 32, 40–43] and another six included patients who were affected by burns [33, 34, 36, 44, 45]. Ten studies (63%) were prospective and the remaining nine were retrospective (37%).

The median number of isolates was 68 (IQR: 405). The largest studies were from Lebanon (2022), Libya (2013) and Iraq (2022) [35–37].

94% of studies considered all sexes and ages, while Dau et al. (Libya, 2011) included exclusively male patients [36]. Eight studies did not specify the distribution of participant sex, [30, 31, 38, 42–47] but in those that did, male populations formed the majority [32–35, 37, 39–41, 48]. In studies that specified, the mean ages of patients varied from 26 to 40 and the range of ages included was from 6 months to 90 years old [30, 32–37, 39, 40, 48]. Patient sample size varied from 99 to 1200 (median 345, IQR = 357), with the largest by Dau et al. [36]. However, seven studies did not detail the number of patients from which isolates were obtained [40–42, 44–47].

### Sampling

Swab samples were used for culture in 13 studies, and 2 studies obtained tissue samples intraoperatively. Four studies did not specify methods of sample collection. The disk diffusion method was used in 15 studies for antimicrobial susceptibility testing, with 13 studies reporting use of CLSI guidelines, two studies reporting use of EUCAST guidelines, [35, 46] one study reporting use of the French Society for Microbiology guidelines [40] and three studies not specifying the guidelines used [30, 32, 39].

Full detail of study characteristics are shown in Table 2.

**Table 2:** Characteristics of studies included in this review, grouped by country, and ordered by publication year

**Study (first author, publication year)**	**Study design**	**Study Period**	**Age (median, unless specified)**	**Sex distribution**		**Type of Injury**	**Number of Isolates/ cultures/ swabs**	**Number Positive Wound Isolates**	**Antibiotic Susceptibility Testing method**	**Guidelines**	**GLASS Pathogens (number of positive isolates)**					**Reference**
				**Male**	**Female**						**SA**	**EC**	**KP**	**PA**	**AB**	
**Iraq**																
** Hateet et al., 2021** [31]	Prospective cross-sectional	October 2020 - May 2021	NS	NS		Burns	105	105	Kirby-Bauer Disc Diffusion	CLSI	18	8	7	21	-	31
** Ali et al., 2024** [34]	Prospective cross-sectional	October 2021 - February 2022	28.03	87	63	Mixed	150	80	Kirby-Bauer Disc Diffusion	CLSI	48	-	-	32	-	34
** M'Aiber et al., 2022** [37]	Retrospective cross-sectional	April 2018 - December 2019	32.6	136	38	Wounds	421	409	Kirby-Bauer Disc Diffusion	CLSI	197	30	22	23	3	37
** Al Miyah et al., 2022** [38]	Prospective cross-sectional	June 2021 - February 2022	NS	NS		Wounds	450	87	Kirby-Bauer Disc Diffusion	CLSI	-	-	87	-	-	38
** Khalid et al., 2024** [41]	Prospective cross-sectional	January 2019 - December 2021	NS	73	44	Burns	NS	117	Disk diffusion method	CLSI	-	-	-	-	117	41
** Aljanaby et al., 2017** [42]	Prospective cross-sectional	July 2016 - January 2017	NS	NS		Burns	144	19	Kirby-Bauer Disc Diffusion	CLSI	-	-	19	-	-	42
** Mahmood et al., 2022** [44]	Prospective cross-sectional	June 2018 to February 2019	NS	NS		Mixed	200	31	Kirby-Bauer Disc Diffusion	CLSI	-	-	-	31	-	44
** Awayid et al., 2022** [45]	Prospective cross-sectional	January - September 2020	NS	NS		Mixed	150	64	Kirby-Bauer Disc Diffusion	CLSI	64	-	-	-	-	45
** Ali et al., 2022** [47]	Retrospective cross-sectional	June 2018-March 2018	NS	NS		Mixed	227	23	VITEK 2	CLSI	-	-	-	23	-	47
**Lebanon**																
** Bourgi et al., 2020** [32]	Retrospective cross-sectional	January 2014 to December 2018	30.3	318	157	Burns	475	261	Kirby-Bauer Disc Diffusion	NS	127	19	18	59	41	32
** Yaacoub et al., 2022** [35]	Retrospective cross-sectional	January 2016 - December 2019	33.5	165	36	Wounds	3204	348	Disk diffusion method	ESCMID/ EUCAST	171	25	18	46	7	35
** Rafei et al., 2015** [40]	Retrospective cross-sectional	2011 - 2013	40	90	26	Wounds	NS	68	Disk diffusion method	French Society of Microbiology	-	-	-	-	70	40
**Libya**																
** Zorgani et al., 2015** [30]	Retrospective cross-sectional	January 2009 - January 2010	Range 2-90	NS		Burns	NS	78	BD geneOhmTM MRSA assay	NS	78	-	-	-	-	30
** Dau et al., 2011** [36]	Prospective cross-sectional	March 2011 - October 2011	30 (mean)	498	0	Mixed	1200	498	Kirby-Bauer Disc Diffusion	CLSI	-	107	86	92	144	36
** Khemiri et al., 2017** [46]	Retrospective cross-sectional	2013	NS	NS		Wounds	32	32	Disc Diffusion	EUCAST	32	-	-	-	-	46
**Yemen**																
** Nasser et al., 2018** [33]	Prospective cross-sectional	January 2017 - April 2017	33.5 (mean)	89	10	Mixed	99	46	Kirby-Bauer Disc Diffusion	CLSI	-	-	-	46	-	33
**Gaza**																
** Elmanama et al., 2013** [43]	Prospective cross-sectional	October 2010 - March 2011	NS	NS		Burns	118	53	Kirby-Bauer Disc Diffusion	CLSI	5	3	1	27	1	43
**Jordan**																
** Teicher et al., 2014** [39]	Retrospective	August 2011-March 2013	26	60	1	Wounds	67	43	MicroScan Walk-Away System	NS	19	8		10	6	39
** Alga et al., 2018** [48]	Retrospective	September 2014-June 2016	27	340	55	Wounds	81	49	VITEK	CLSI	15	8	11	12	5	48

*SA* S. aureus, *KP* K. pneumoniae, *EC* E. coli, *PA* P. aeruginosa, *AB* A. baumannii, *NS* not specified, *CLSI* Clinical and Laboratory Standards Institute. *EUCAST* European Committee on Antimicrobial Susceptibility Testing.

## Quality assessment

Using the Joanna Briggs checklists, and pre-defined scoring methods, nine studies were graded as carrying a high risk of bias, [30, 31, 39–42, 44–46] six as carrying a moderate risk of bias, [32–34, 36, 38, 47] and only four as carrying a low risk of bias [35, 37, 43, 48].

## Isolated organisms

**Fig. 3:**
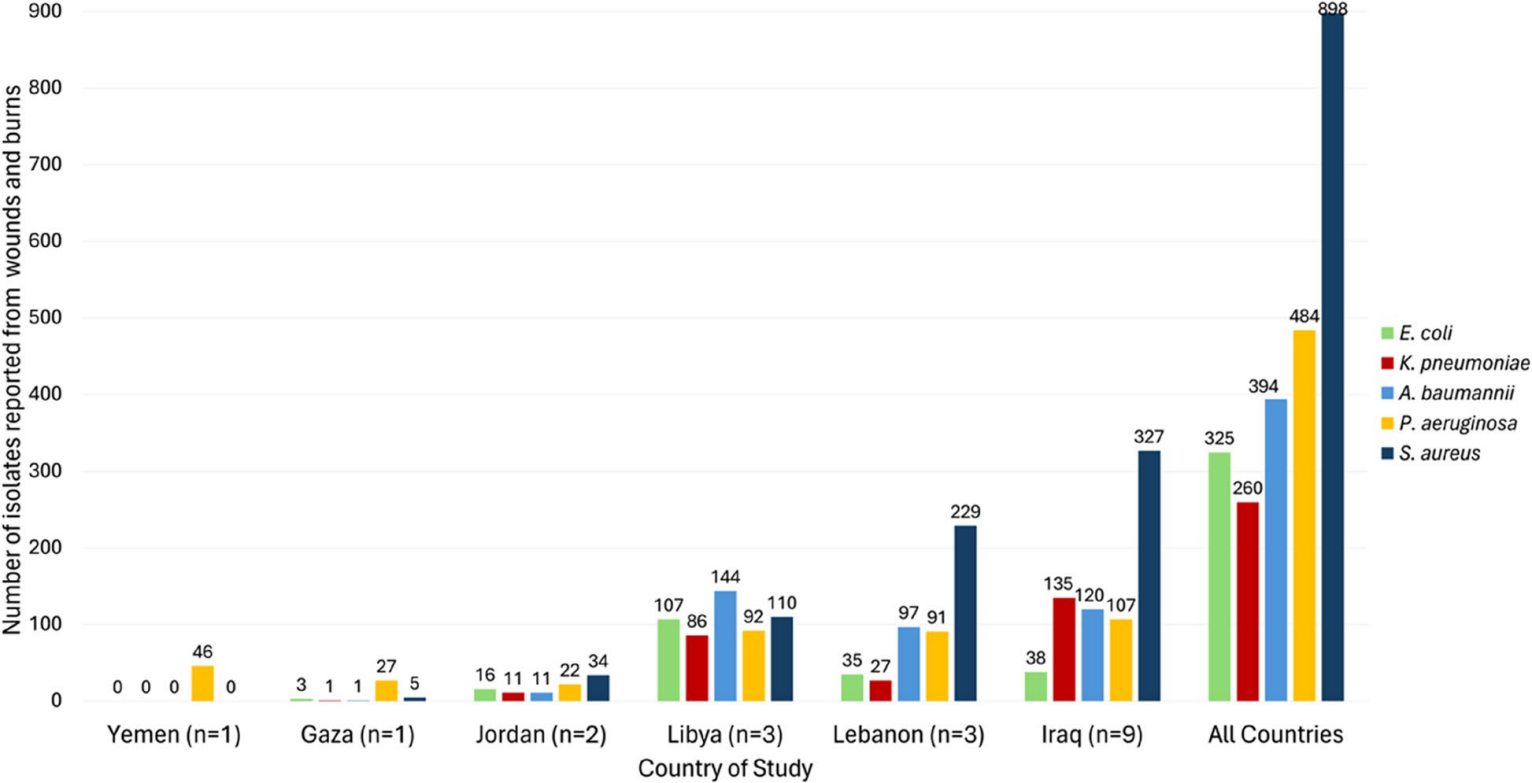
Number of positive wound and burn isolates identified for pathogens of interest in this review. A graph displaying the total number of positive wound and burn isolates identified for each Global Antimicrobial Resistance Surveillance and Use System priority pathogen from the studies in this review. Data grouped according to country. n = number of studies

The most prevalent GLASS pathogen reported was *S. aureus* comprising 36.3% (705/1942) of the total isolates reported from all studies. This was followed by *P. aeruginosa* (19.8%, n = 385) and *A. baumannii* (19.2%, n = 373). Figure 3 displays the total number of positive isolates reported from each country.

Gram positive isolates (n = 924) were discussed in eleven studies, ten of which reported *S. aureus.* Dau et al. reported only coagulase negative staphylococci so these were not included [36]. The combined total of *S. aureus* isolates was 705, with the largest studies from 2022 in Lebanon (n = 171) and Iraq (n = 197) which accounted for 24.3% and 27.9% of all *S. aureus* isolates reported [35, 37]. Of these, ten studies reported findings regarding MRSA. Eight of these calculated the proportion of *S. aureus* that were MRSA, while two studies from Libya only included MRSA isolates in their results [30, 32, 34–36, 39, 43, 45, 46, 48].

Five studies assessed vancomycin susceptibility in *S. aureus* isolates. The results are shown in Table 3*.* Within these studies, four report no resistance to vancomycin among *S. aureus* isolates, and one study reported 2.7% vancomycin resistance. This raises concern for rare vancomycin-intermediate or vancomycin-resistant *S. aureus* (VRSA) isolates. However, the study does not detail any subsequent testing, such as an Epsilometer test, that took place to confirm these results [45].

**Table 3:** Reported results of resistance rates of *Staphylococcus aureus* to methicillin and vancomycin

Study	Country of Study	Number of Isolates	Methicillin Resistant *S. aureus* (%)	Vancomycin resistance (%)
Elmanama, 2013 [43]	Gaza	5	60	-
Bourgi, 2020 [32]	Lebanon	127	100	-
Yaacoub, 2022 [35]	Lebanon	171	48.5	0
M’aiber, 2022 [37]	Iraq	197	94.4	0
Ali, 2024 [34]	Iraq	48	33.3	-
Awayid, 2022 [45]	Iraq	64	53.1	2.7
Zorgani, 2015 [30]	Libya	78	100*	0
Khemiri, 2017 [46]	Libya	32	100*	0
Teicher, 2014 [39]	Jordan (Syria)	19	42	-
Alga, 2018 [48]	Jordan (Syria)	49	73	-
Median Values (IQR)			56.55% (49.65–90.3)	0% (0–1.35)

*Studies ordered primarily by country of publication and secondarily by year of publication. Studies that included only methicillin resistant* S. aureus isolated in their analysis marked by an asterisk (*), and these values were excluded from median calculations

**Fig. 4:**
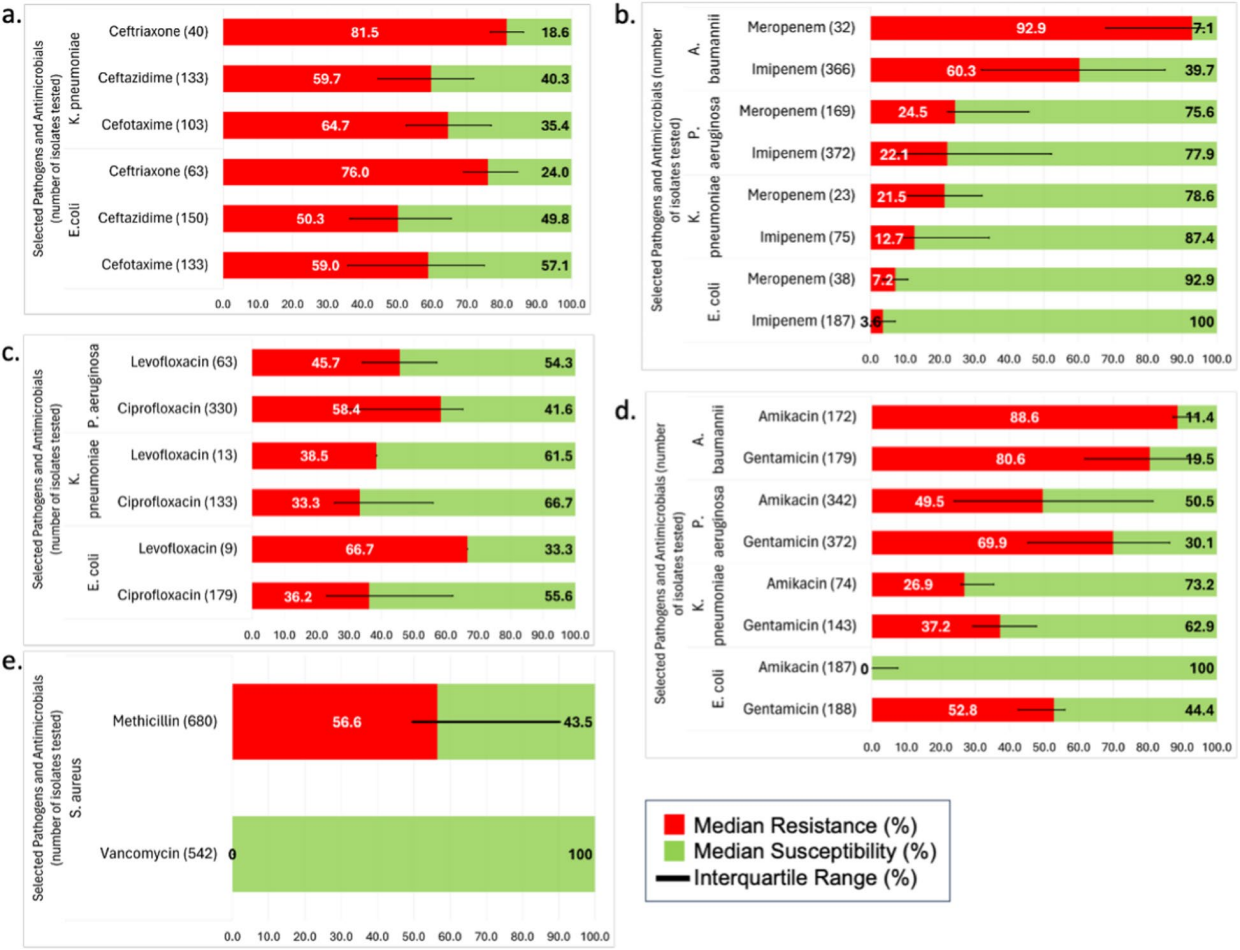
Median resistances of Global Antimicrobial Resistance and Use Surveillance System priority pathogens to selected antimicrobials. Calculated median resistances and susceptibilities of Global Antimicrobial Resistance and Use Surveillance System priority pathogens to: 4a. selected third generation cephalosporins; 4b. selected carbapenems; 4c. selected fluoroquinolones; 4d. selected aminoglycosides; 4e. resistances of *S. aureus* to methicillin and vancomycin

Gram negative rods (GNRs) were discussed in 84% of studies (16/19) with a total of 1482 reported. Median resistance rates to antimicrobial classes are displayed in Fig. 4*.* Proportion of ESBL-producing GNRs were reported in three studies [37, 42, 47]. M’aiber et al. (Iraq, 2022) studied 349 wound patients and reported 28 ESBL-producing *E. coli* (93.3%), and 20 ESBL-producing *K. pneumoniae* isolates (90.9%) [37]. Aljanaby et al. (Iraq, 2017) studied burns patients finding 19 (100%) of ESBL-producing *K. pneumoniae* isolates [42]. Ali et al. (Iraq, 2022) reported on ESBL-producing *P. aeruginosa* isolates, finding nine (100%) isolates from wounds, and ten (71.4%) from burns [47].

Carbapenem resistant *Enterobacterales* (CREs) were reported in ten studies across all countries except Yemen. Elmanama et al. (Gaza, 2013) reported an Imipenem resistance rate of 20% among *Enterobacterales* isolates [43]. Alga et al. reported that among isolates of *E. coli* (n = 8, 100%) and *K. pneumoniae* (n = 11, 100%) were susceptible to carbapenems [48]. Hateet et al. (Iraq, 2021), in a study of 105 patients, reported the highest rates of CREs [31]. Resistance to imipenem was 66.7% (n = 8) among *K. pneumoniae* isolates, and 100% (n = 7) among *E. coli* isolates. Across the remaining seven studies, a collective 241 isolates of *K. pneumoniae*, and 183 isolates of *E. coli* were tested. Resistance to imipenem was 11% (IQR:9.7–34.3%) for *K. pneumoniae* isolates, and no resistance (0%, IQR:0–12.23%) for *E. coli* isolates. Only two studies tested resistance to meropenem, with one reporting resistance in 14.3% of *E. coli* isolates and 43% of *K. pneumoniae* isolates, [37] and the other reporting no resistance in both *E. coli* and *K. pneumoniae* isolates [32].

For individual GNRs, *P. aeruginosa* was reported in eleven studies across all six countries, with the largest sample reported from Libya (n = 92) [36]. All other studies reported sample sizes less than 50 isolates. From the total of 372 isolates tested, the highest resistance was to gentamicin with a median of 69.94% (IQR:45.25–86.35%). Resistance rates to amikacin, ciprofloxacin and levofloxacin were all above 40%, although only two studies included resistances to levofloxacin [36, 44]. Carbapenem resistance among Pseudomonal isolates was lower, with resistance to imipenem in 22.14% (IQR:7.43–52.22%) of isolates and resistance to meropenem in 24.45% (IQR:22.25–45.7%) of isolates.

A total of 373 *A. baumannii* isolates were identified across nine studies, with the largest number of samples reported from Libya (n = 144; 38.6%) [36]. Five studies had sample sizes with fewer than ten isolates, and the remaining studies had a moderate or high risk of bias [32, 35–37, 39–41, 43, 48]. Elmanama et al. did not report resistance rates, while Alga et al. reported only that most isolates were carbapenem resistant [43]. Across all other studies, median carbapenem resistance rates were: 60.3% (IQR:32.1–85%) to imipenem, and 92.9% (IQR:67.9–96.5%) to meropenem. The highest values were reported by Bourgi et al. (90% resistance to Imipenem and 92.9% to meropenem) and M’aiber et al. (100% resistance to Imipenem and Meropenem) [32, 37]. Aminoglycoside resistance rates were reported in four studies. Median proportion of resistance to gentamicin was 80.55% (IQR:61.73–94.83%), compared to 88.6% (IQR:87.3.6–94.3%) for amikacin, although only three studies assessed both agents [32, 35, 36, 39].

Multidrug resistance was explicitly reported by eleven studies, most of which followed the Magiorakos et al. definition of MDR [17]. Across all pathogens isolated in M’aiber et al., overall MDRO prevalence was 86% (n = 352), representing 81% of patients [37]. Where reported, prevalence of MDR *K. pneumoniae* was high, ranging from 42.1% (with an additional 47.4% being extensively drug resistant) to 90.9% [35, 37, 38, 42, 48]. MDR *P. aeruginosa* varied from 7.6% to 100%, with no obvious correlation to country or year [33, 35, 43, 44, 48]. Two studies located in Lebanon reported MDROs [32, 35]. Yaacoub et al. found 55.7% (186/334) of isolates were MDROs, with no significant differences between age or gender [35]. Amongst all *Enterobacterales* isolates, over 80% of isolates were MDROs. The authors reported there was no significant association between MDRO and the year of sampling. Bourgi et al. identified that most isolates from their cohort of infected burn patients were either extensively- or multi-drug resistant, including *A. baumannii*, *S. aureus* and *P. aeruginosa* [32]. Similarly, a study carried out in Jordan, identified that 100% of *E. coli* and *A. baumannii* isolates, and 82% of *K. pneumoniae* isolates, were MDR [48]. Further detail on these results, including definitions used in each study, are in *Additional file 2.2.*

## Iraq

Nine studies were carried out in Iraq. Three considered solely burns, two considered only wounds, and the remaining four comprised a combination of burns and wounds [31, 34, 37, 38, 41, 42, 44, 45, 47]. Three studies compared multiple pathogens, finding the most prevalent were either *S. aureus* or *P. aeruginosa* [31, 34, 37]. M’aiber et al. had the largest sample size of positive wound isolates (n = 349;39.9%), 81% of which were isolated from patients with violent traumatic injuries [37]. This was the only study from Iraq with a low risk of bias score.

Four studies identified resistance rates to third generation cephalosporins (3GCs) [31, 37, 38, 42]. Resistance of 135 K*. pneumoniae* isolates was reported [31, 37, 38, 42]. Resistance rates to 3GCs (cefotaxime, ceftazidime, ceftriaxone) in all studies were over 50% for *K. pneumoniae* isolates. Aljanaby et al. identified 19 strains of *K. pneumoniae* from 92 burns swabs, with high resistances to 3GCs, ranging from 90.69–96.67% [42]. *E. coli* resistance to 3GCs was only reported in two studies, with a total of 38 isolates [31, 37]. M’aiber et al. found 28 *E. coli* isolates (93.3%) were resistant to ceftriaxone, while Hateet et al. reported six *E. coli* isolates were resistant to cefotaxime (14.3%) [31, 37].

## Lebanon

Three studies were carried out in Lebanon [32, 35, 40]. Yaacoub et al. reported three years of data and had the largest GLASS pathogen isolate number in Lebanon (n = 267, 55.7%) [35]. Two studies included multiple pathogen types, and reported *S. aureus* was the most prevalent pathogen, followed by *P. aeruginosa* [32, 35]. Yaacoub et al. reported no significant difference in the type of pathogen identified in relation to year of collection, nationality or sex [35]. Bourgi et al. identified 144 positive GLASS pathogen isolates, of which *S. aureus* was the most prevalent (n = 58, 40.2%). This was the only study in Lebanon to consider burns [32].

Only two studies considered resistance to 3GCs [32, 35]. Yaacoub et al. reported resistance of *K. pneumoniae* isolates was 72% to ceftazidime and ceftriaxone, while 75% of *E. coli* isolates were resistant to these antimicrobials [35]. Bourgi et al. reported resistance rates of 44.4% and 53.4% for the nine *K. pneumoniae* isolates tested to cefotaxime and ceftazidime [32]. This study identified ten *E. coli* isolates and found resistances of 42.9% and 38.5% to cefotaxime and ceftazidime respectively.

## Libya

Three studies from Libya were included in this review, two of which studied only *S. aureus*, with a combined sample size of 110 [30, 36, 46]. Dau et al. published a high- quality study with a sample size of 429 isolates from both wound and burn swabs [36]. This is the only study in which *Acinetobacter* was the most prevalent pathogen (n = 144, 33.6%). In this study, 86 K*. pneumoniae* isolates (100%) and 107 *E. coli* isolates (100%) were susceptible to imipenem. Dau et al. also reported resistances to 3GCs. 32 (30%) *E. coli* isolates and 13 (15%) of *K. pneumoniae* isolates were resistant to ceftazidime. Cefotaxime resistance was reported as 65 K*. pneumoniae* isolates (76%) and 80 *E. coli* isolates (75%) [36].

## Jordan

Two retrospective studies included patients from conflict affected settings who were treated in Jordan. Teicher et al. carried out a study in Amman, Jordan, and identified that 18% (61/345) of Syrian patients who underwent surgical procedures had a suspected infection [39]. Of the eight confirmed *E. coli* isolates from these patients, resistance rates to 3GCs varied between 62–75%.

Alga et al. similarly identified clinical signs of infection in 81/457 (18%) Syrian patients, from which 49 positive isolates were identified [48]. Among these, it was reported that of the 49 K. pneumoniae and 49 *E. coli* isolates, resistance to most antimicrobials was observed, except for carbapenems, although exact resistance values were not given [48].

## Other countries

Nasser et al. was the only study located in Yemen [33]. In that study, 44 *P. aeruginosa* isolates were identified from conflict-related wounds, 65.2% (n = 30) of which were identified as MDRO. These isolates showed a resistance rate to imipenem of 19.6% and a resistance rate to gentamicin of 87%.

One study was located in Gaza. Elmanama et al. described 53/118 (44.9%) burn and wound swabs resulted were positive, with *Pseudomonas* and *Enterobacter* species accounting for 53% of isolates [43]. Among the samples, 70% of *Enterobacteriaceae* were resistant to ceftriaxone which was the only 3GC tested.

## Discussion

This review raises concern for a high burden of MDRO-attributed infections among civilians with conflict-associated wounds or burns in the Eastern Mediterranean. Specific pathogens with concerning rates of resistance are MRSA, 3GC-resistant *Enterobacterales*, and carbapenem-resistant *A. baumannii* (CRAB). However, due to impaired study quality, methodological heterogeneity, and no representation from five of the ten eligible countries, more research is required to establish the true burden of conflict-associated AMR within this region.

*Enterobacterales*, specifically *E. coli* and *K. pneumoniae*, showed high rates of resistance to 3GCs in this review, with the highest rates to ceftriaxone, one of the most clinically utilised of this class [49]. Literature from non-conflict EMRO countries have identified similar findings. Fily et al. studied Syrian, Iraqi and Yemeni patients (also treated in Jordan) with suspected post-traumatic infection and reported that *Enterobacterales* resistance to 3GCs remained above 80% across nine years [50].

Almehdar et al. reported on 481 conflict-injured patient who were admitted to a Medecins Sans Frontieres hospital in Yemen and prescribed antibiotics [51]. Among these infections, 268 were due to osteomyelitis or skin and soft tissue infections. 85.2% of *E. coli* isolates and 80.6% of *K. pneumoniae* isolates were identified as resistant to ceftriaxone. Both reports corroborate the findings of highly resistant *E. coli* and *K. pneumoniae* to 3GCs in this study. *P. aeruginosa* isolates (n = 385) were the 2nd most prevalent, and had variable MDR rates (7.6–100%), median imipenem resistance: 22%.

Furthermore, high rates of MRSA were identified in this review. The high IQR and maximum values of 100% suggest a concerning prevalence of MRSA across all countries considered. Reports of high proportions of MRSA isolates from Fily et al., Almehdar et al. and Murphy et al. (60.5%, 72% and 84% respectively) among populations originally from Syria, Yemen and Iraq reflect the MRSA proportions observed in this review [50–52]. In addition, a concerning report of possible VRSA was identified in this review [44]. While this finding was not appropriately confirmed, only four other studies assessed vancomycin susceptibility within this population. Given the high risks associated with VRSA, this could highlight an area for further study.

A high proportion of CRAB was also identified as MDR in this review. The prevalence of *A. baumannii* was variable across studies, and often represented a small proportion of total isolates identified. Nonetheless, the continued presence of *A. baumanii* isolates and high rates of resistance among this population suggest it is an ongoing concern, in-keeping with existing literature. Almehdar et al. published a study after the search date of this review, identifying 87 *A. baumannii* isolates from Yemeni patients (2018–2021) [51]. In that study, 96.5% of *A. baumannii* isolates were carbapenem-resistant, comparable to the high median identified in this review. Further, a study in Lebanon of infected civilians identified a marked decrease of *Acinetobacter* species susceptibility to imipenem, from 49 to 15% across a three-year period, while Truppa et al. found an overall increase in imipenem resistance of *Acinetobacter* from 2012–2018 across all EMRO countries [16, 53]. Among wounded military personnel, CRAB has been associated with osteomyelitis, increased length of stay, intensive care unit admission and higher mortality rates among the immunocompromised [54]. Despite small sample sizes in this review, the existing evidence presented supports the findings of this review and indicate CRAB presents a threat to wounded EMRO civilians.

Whilst this review found a strong indication of MDROs among wounded civilians, studies from only five of the ten eligible locations met inclusion criteria, with the majority originating from Iraq. The frequent inclusion of Iraq, could be due to the historical association of conflicts in Iraq with MDROs, particularly *A. baumannii* [55–58]. Alternatively, as Iraq is now a “post-conflict state”, the more stable environment in comparison to other countries may have facilitated research [59]. This is supported by the fact that eight of the nine studies from Iraq in this review were published after 2022. The geographic skew observed means certain eligible countries are not represented in this study, an issue that is common among existing literature [16, 60].

The deficit of data from certain conflict affected EMRO countries in this review is concerning as the prevalence of AMR remains undetermined. While there is evidence that limited AMR data has been collected from within these countries, it fails to robustly examine wounded civilians. A review from Syria (2018) identified a growing prevalence of resistant Gram-negative organisms and MRSA among patients with urinary tract, respiratory and other non-wound related infections [12]. It was highlighted that, despite the extensive conflict and 1.9 million Syrians having been wounded or injured, there was a dearth of data regarding war-related injuries, including wounds and burns. The current review has identified that no literature regarding wounded civilians has been published in the subsequent years in Syria. In other countries, a study from Somalia identified that among uropathogens, 69.1% of *A. baumannii* and 35.2% of *E. coli* and *K. pneumoniae* isolates were phenotypically MDROs [61]. In Djibouti, Ragueh et al. recently reported that 95.7% of clinical isolates (including *Enterobacterales* and *A. baumannii*) were MDRO [62]. These findings indicate that reporting AMR data from within conflict affected EMRO countries is possible. They also emphasise that wounded civilians are at risk of infection with MDR GLASS pathogens. As such, data collection of AMR among conflict-wounded civilians within the EMRO should be prioritised in order to establish an accurate burden of AMR.

This is the first review to address the resistance patterns of GLASS pathogens isolated from conflict-associated wounds and burns among civilians in EMRO countries. The information presented is up to date regarding MDROs within this specific population and focused on recently updated GLASS Priority Pathogens (2024) [19]. An extensive time period was considered within this review. This encompassed prominent conflicts within the EMRO, and captured data from the time of conflict, or the aftermath in which civilians face long-term healthcare consequences. Such recent data provides understanding of how MDROs affect civilians wounded in conflict and is timely given the recent escalation of violence in Sudan, Yemen, and Gaza [63–65]. This review highlights the need for greater data collection in these conflicts and can provide valuable information for future antibiotic protocols directed towards the wounded civilians.

This review has certain limitations. Firstly, studies from non-conflict affected countries were excluded from this review, but patients from the population of interest have been treated in these countries. This has resulted in an excluded cohort of eligible wounded civilians, leaving their pathogen and AMR profiles unincorporated. Noting such, we identify a population of focus for future research, particularly as nearly 68% of refugees globally are from EMRO countries including Somalia, Syria and Afghanistan [66].

Secondly, some papers included were vague regarding patient characteristics. This has been considered in the quality assessment of these papers and embedded in the inclusion and exclusion criteria of this review. Nevertheless, it remains possible that data from some patients assessed in this review do not truly fit the definition of “conflict-associated wounds”. Although careful assessment of context and methodology was undertaken prior to study inclusion to minimise this risk, such errors could reduce the accuracy of the aforementioned results. In particular, there is a lack of detail as to the interactions between civilians and military personnel e.g. if there are military family members which could result in the transfer to resistant organisms from military to civilians in the same household. Additionally, in certain conflicts, particularly besieged areas, there is likely to be a great deal of mixing in both community and health facilities which will be accessed by the whole population. However, our aim in this review is to capture the main resistance patterns which are evident and to make the distinction between ‘international’ military personnel compared to local civilians in particular. To fully address these nuances, ideally, prospective data collection where such characteristics are detailed in real time, would be needed.

Finally, the nature of research within conflict-affected situations presents an inherent limitation regarding the reliability of the data. As the severity of conflicts change, so too do the challenges surrounding the administration, risk management and priorities of healthcare workers [67]. Data collection may be disregarded for extended periods of time. The extent of any such inconsistencies, and their effects on the results, are unknown.

## Implications and future research

High levels of MDRO-associated infections among civilian populations with wounds and burns promotes the need for effective infection prevention and control protocols to be implemented within conflict-affected countries as well as those hosting displaced communities. Unstable environments and emergency situations pose unique challenges in implementing these protocols, but simplification and adaptation to the environment can enable their success in mitigating AMR [3]. Concurrent antimicrobial stewardship should also be prioritised in accordance with the Global Action Plan for tackling AMR [2]. Such stewardship should focus on informed prescribing rather than empirical treatment. This is particularly pertinent for broad, high-risk antimicrobials, such as carbapenems, to minimise selective pressures which could drive future resistance. Cohesive, national policies that enforce regulations, and training on these protocols, are crucial for successful implementation.

Finally, surveillance is critical to accurately assess the burden and evolution of AMR. Effective surveillance includes consistent documentation of specified clinical and patient parameters, and laboratory confirmed microbiological and susceptibility data. However, heterogeneity across data acquisition methods, as identified within this review, has been an issue with AMR data from the EMRO. For example, this review found inconclusive evidence for longitudinal trends due to inconsistencies in methodology, particularly in the reporting of pathogen-antibiotic relationships across multiple years. Homogeneous surveillance data will facilitate the generation of high quality, large scale and prospective studies which are essential to gain an accurate representation of the current AMR burden. Improvements in primary data could facilitate better understanding of epidemiological risk factors of MDRO infections, such as mechanism of injury, age, or other patient characteristics. It could also enable the evaluation of policy impacts, and identify quality improvement initiatives for clinical care, such as the compilation of informed antimicrobial use guidelines. Surveillance should be prioritised at national and local levels within all conflict-affected countries in the EMRO, including those that are currently under-represented, and specifically regarding wounded civilians. Additionally, there needs to be a stronger emphasis on the strengthening and quality of microbiological performance such that the required diagnostics, skilled staff and quality control measures are in place to ensure robust data on which to base recommendations. This also requires standardization of diagnostics for the susceptibility patterns and the mechanisms of resistance were identified; this will improve the ability to track resistance and make comparisons across contexts.

## Conclusion

This review indicates high prevalence of MDR GLASS pathogens within conflict-affected countries within the Eastern Mediterranean. It also identifies that wounded civilian populations continue to be under-researched, with existing evidence being scarce and of suboptimal quality. Addressing the scale of the AMR issue is challenging due to a lack of primary data, suggesting that the current burden is likely underreported. The threat of AMR continues to evolve, and policy, intervention, and clinical treatment must adjust accordingly. Effective surveillance that establishes accurate data on prevalence and trends across time, regions and populations is essential to guide future coordinated responses to AMR.

## Supplementary Information

Supplementary Material 1.

Supplementary Material 2.

## Data Availability

Definitions used in this review, the search strategy, quality assessment and data extraction prioritised for understanding are available in Supplementary Materials. Complete data extraction are available from the corresponding author under reasonable request.

## Abbreviations

WASH: Water, sanitation and hygiene
WHO: World Health Organisation
AMR: Anti-microbial resistance
GLASS: Global Antimicrobial Surveillance System
MRSA: Methicillin resistant *Staphylococcus aureus*
MDRO: Multi-drug resistant organism
MDR: Multi-drug resistant
EMRO: World Health Organisation Eastern Mediterranean Region
IQR: Interquartile range
GNR: Gram negative rods
3GCs: Third generation cephalosporins
CRAB: Carbapenem-resistant *Acinetobacter baumannii*
VRSA: Vancomycin resistant *Staphylococcus aureus*

## References

[1] Comelli A, Gaviraghi A, Cattaneo P, Motta L, Bisoffi Z, Stroffolini G. Antimicrobial Resistance in Migratory Paths, Refugees, Asylum Seekers and Internally Displaced Persons: A Narrative Review. Curr Trop Med Rep. 2024;11(3):153–66.

[2] Naim C, Pokorny J, Uyen A, Shortall C, Farra A, Moussally K. The role of humanitarian actors in global governance for AMR. Lancet Glob Health. 2024;12(11):e1752–3. 10.1016/S2214-109X(24)00319-X. 39074476 10.1016/S2214-109X(24)00319-X

[3] Pallett SJC, Boyd SE, O’Shea MK, Martin J, Jenkins DR, Hutley EJ. The contribution of human conflict to the development of antimicrobial resistance. Communications Medicine. 2023;3(1):153. 37880348 10.1038/s43856-023-00386-7 PMC10600243

[4] Abbara A, Rawson TM, Karah N, El-Amin W, Hatcher J, Tajaldin B, et al. Antimicrobial resistance in the context of the Syrian conflict: Drivers before and after the onset of conflict and key recommendations. Int J Infect Dis. 2018;73:1–6. 29793039 10.1016/j.ijid.2018.05.008

[5] Fouad FM, Sparrow A, Tarakji A, Alameddine M, El-Jardali F, Coutts AP, et al. Health workers and the weaponisation of health care in Syria: a preliminary inquiry for The Lancet –American University of Beirut Commission on Syria. The Lancet. 2017;390(10111):2516–26. 10.1016/S0140-6736(17)30741-9 28314568

[6] Médecins Sans Frontières. MSF mourns the killing of our colleague in Gaza. https://msf.org.uk/article/msf-mourns-killing-our-colleague-gaza. Accessed 26 Sep 2024.

[7] Shallal A, Lahoud C, Zervos M, Matar M. Antibiotic Stewardship in Disaster Situations: Lessons Learned in Lebanon. Antibiotics. 2022;11(5):560. 35625204 10.3390/antibiotics11050560 PMC9137475

[8] World Bank. FY06–23 List of Fragile and Conflict-affected Situations.[internet]. 2023 Jul. Accessed 22 May 2024. Available from: https://www.worldbank.org/en/topic/fragilityconflictviolence/brief/harmonized-list-of-fragile-situations.

[9] World Health Organisation. World Health Organisation Eastern Mediterranean Region - Countries. [internet]. Accessed 22 May 2024. Available from: https://www.emro.who.int/countries.html.

[10] Rex B. Beyond the Arab spring : authoritarianism & democratization in the Arab world. Boulder: Lynne Rienner Publishers; 2012.

[11] Moussally K, Abu-Sittah G, Gomez FG, Fayad AA, Farra A. Antimicrobial resistance in the ongoing Gaza war: a silent threat. The Lancet. 2023;402(10416):1972–3. 10.1016/S0140-6736(23)02508-4 37952545

[12] Abbara A, Rawson TM, Karah N, El-Amin W, Hatcher J, Tajaldin B, et al. A summary and appraisal of existing evidence of antimicrobial resistance in the Syrian conflict. Int J Infect Dis. 2018;75:26–33. 29936319 10.1016/j.ijid.2018.06.010

[13] Abbara A, Al-Harbat N, Karah N, Abo-Yahya B, El-Amin W, Hatcher J, et al. Antimicrobial Drug Resistance among Refugees from Syria. Jordan Emerg Infect Dis. 2017;23(5):885–6. 28418320 10.3201/eid2305.170117 PMC5403049

[14] World Health Organisation Eastern Mediterranean Region. Statement by the Regional Director at the World Antimicrobial Resistance Awareness Week 2023 press conference. 2023 [cited 2025 Jan 31]; [1]. Available from: https://www.emro.who.int/media/news/statement-by-the-regional-director-at-the-world-antimicrobial-resistance-awareness-week-2023-press-conference.html.

[15] Global Database for Tracking Antimicrobial Resistance (AMR) Country Self- Assessment Survey (TrACSS). TrACSS Country Reports. 2022. Accessed 22 May 2024. Available from: https://www.who.int/teams/surveillance-prevention-control-AMR/national-action-plan-monitoring-evaluation/tracss-2022-country-profiles.

[16] Truppa C, Abo-Shehada MN. Antimicrobial resistance among GLASS pathogens in conflict and non-conflict affected settings in the Middle East: a systematic review. BMC Infect Dis. 2020;20(1):936. 33297983 10.1186/s12879-020-05503-8 PMC7724697

[17] Magiorakos AP, Srinivasan A, Carey RB, Carmeli Y, Falagas ME, Giske CG, et al. Multidrug-resistant, extensively drug-resistant and pandrug-resistant bacteria: An international expert proposal for interim standard definitions for acquired resistance. Clin Microbiol Infect. 2012;18(3):268–81. 21793988 10.1111/j.1469-0691.2011.03570.x

[18] Friedman ND, Temkin E, Carmeli Y. The negative impact of antibiotic resistance. Vol. 22, Clinical Microbiology and Infection. Elsevier B.V.; 2016. p. 416–22. 10.1016/j.cmi.2015.12.002 26706614

[19] World Health Organisation. WHO bacterial priority pathogens list, 2024: Bacterial pathogens of public health importance to guide research, development and strategies to prevent and control antimicrobial resistance. Geneva: World Health Organisation; 2024 [cited 2025 Jan 31]. 72p. ISBN:978-92-4-009346-1. Licence: CC BY-NC-SA 3.0 IGO. Available from: https://www.who.int/publications/i/item/9789240093461.

[20] Boukari Y, Kadir A, Waterston T, Jarrett P, Harkensee C, Dexter E, et al. Gaza, armed conflict and child health. BMJ Paediatr Open. 2024;8(1):e002407. 38350977 10.1136/bmjpo-2023-002407 PMC10868171

[21] Devi S. AMR in the Middle East: “a perfect storm.” Lancet. 2019;394(10206):1311–2. 31609216 10.1016/S0140-6736(19)32306-2

[22] Chandrasekera RM, Lesho EP, Chukwuma U, Cummings JF, Waterman PE. The state of antimicrobial resistance surveillance in the military health system: A review of improvements made in the last 10 years and remaining surveillance gaps. Military Medicine. Association of Military Surgeons of the US. 2015;180:145–50. 10.7205/MILMED-D-14-00297 25643381

[23] PRISMA. Preferred reporting items for systematics reviews and meta-analyses: PRISMA 2020 checklist. 2020. Accessed 22 May 2024. Available from: https://www.prisma-statement.org.

[24] Operational Data Portal. Syria Regional Refugee Response. Available at: https://data.unhcr.org/en/situations/syria. Accessed 26 Sep 2024.

[25] Church D, Elsayed S, Reid O, Winston B, Lindsay R. Burn wound infections. Clin Microbiol Rev. 2006;19:403–34. 16614255 10.1128/CMR.19.2.403-434.2006 PMC1471990

[26] Giacometti A, Cirioni O, Schimizzi AM, Del Prete MS, Barchiesi F, D’errico MM, et al. Epidemiology and Microbiology of Surgical Wound Infections. J Clin Microbiol. 2000;38(2):918–22. 10655417 10.1128/jcm.38.2.918-922.2000 PMC86247

[27] Checklist for Analytical Cross Sectional Studies Critical Appraisal Checklist for Analytical Cross Sectional Studies 2. 2017. Accessed 22 May 2024. Available from: http://joannabriggs.org/research/critical-appraisal-tools.htmlwww.joannabriggs.org.

[28] Nasher S, Alsharapy S, Al-Madhagi A, Zakham F. Epidemiology of extended-spectrum β-lactamase producing escherichia coli from hospital settings in yemen. J Infect Dev Ctries. 2018;12(11):953–9. 32012124 10.3855/jidc.10560

[29] Hussein NH, Mohammed Kareem S, Hussein AL-Kakei SN, Taha BM. The predominance of Klebsiella pneumoniae carbapenemase (KPC-type) gene among high-level carbapenem-resistant Klebsiella pneumoniae isolates in Baghdad. Iraq Mol Biol Rep. 2022;49(6):4653–8. 35471622 10.1007/s11033-022-07314-3

[30] Zorgani AA, Elahmer O, Abaid A, Elaref A, Elamri S, Aghila E, et al. Vancomycin susceptibility trends of methicillin-resistant staphylococcus aureus isolated from burn wounds: A time for action. J Infect Dev Ctries. 2015;9(11):1284–8. 26623639 10.3855/jidc.6976

[31] Hateet RR. Isolation and Identification of Some Bacteria Contemn in Burn Wounds in Misan. Iraq Arch Razi Inst. 2021;76(6):1665–70. 35546990 10.22092/ari.2021.356367.1833 PMC9083866

[32] Bourgi J, Said JM, Yaakoub C, Atallah B, Al Akkary N, Sleiman Z, et al. Bacterial infection profile and predictors among patients admitted to a burn care center: A retrospective study. Burns. 2020;46(8):1968–76. 32522390 10.1016/j.burns.2020.05.004

[33] Nasser M, Ogali M, Kharat AS. Prevalence of MDR Pseudomonas aeruginosa of war-related wound and burn ward infections from some conflict areas of Western Yemen. Wound Medicine. 2018;1(20):58–61.

[34] Ali S, Assafi M. Prevalence and antibiogram of Pseudomonas aeruginosa and Staphylococcus aureus clinical isolates from burns and wounds in Duhok City. Iraq J Infect Dev Ctries. 2024;18(1):82–92. 38377094 10.3855/jidc.18193

[35] Yaacoub S, Truppa C, Pedersen TI, Abdo H, Rossi R. Antibiotic resistance among bacteria isolated from war-wounded patients at the Weapon Traumatology Training Center of the International Committee of the Red Cross from 2016 to 2019: a secondary analysis of WHONET surveillance data. BMC Infect Dis. 2022;22(1):257. 35287597 10.1186/s12879-022-07253-1 PMC8922823

[36] Dau AA, Tloba S, Daw MA. Characterization of wound infections among patients injured during the 2011 Libyan conflict. East Mediterr Health J. 2013;19(4):356-61. 23882961

[37] M’Aiber S, Maamari K, Williams A, Albakry Z, Taher AQM, Hossain F, et al. The challenge of antibiotic resistance in post-war Mosul, Iraq: an analysis of 20 months of microbiological samples from a tertiary orthopaedic care centre. J Glob Antimicrob Resist. 2022;1(30):311–8. 10.1016/j.jgar.2022.06.022 35768065

[38] Fazaa A, ALmiyah S. Detection of AcrA and AcrB Efflux Pumps in Multidrug-Resistant Klebsiella pneumonia that Isolated from Wounds Infection Patients in Al-Diwaniyah Province. Arch Razi Inst. 2023;78(1):263–70. 10.22092/ARI.2022.358956.2342 PMC10258248 37312720

[39] Teicher CL, Ronat JB, Fakhri RM, Basel M, Labar AS, Herard P, et al. Antimicrobial Drug-Resistant Bacteria Isolated from Syrian War-Injured Patients, August 2011–March 2013. Emerg Infect Dis. 2014;20(11):1949–51. 25340505 10.3201/eid2011.140835 PMC4214314

[40] Rafei R, Pailhoriès H, Hamze M, Eveillard M, Mallat H, Dabboussi F, et al. Molecular epidemiology of Acinetobacter baumannii in different hospitals in Tripoli, Lebanon using bla OXA-51-like sequence based typing. BMC Microbiol. 2015;15(1):103. 25976451 10.1186/s12866-015-0441-5 PMC4432822

[41] Khalid HM. Molecular study of blaVIM and blaIMP genes in Acinetobacter baumannii strains isolated from burn patients in Duhok City. Iraq J Infect Dev Ctries. 2024;18(1):101–5. 38377096 10.3855/jidc.18570

[42] Aljanaby AAJ, Alhasnawi HMRJ. Phenotypic and molecular characterization of multidrug resistant Klebsiella pneumoniae isolated from different clinical sources in Al-Najaf Province-Iraq. Pak J Biol Sci. 2017;20(5):217–32. 29023034 10.3923/pjbs.2017.217.232

[43] Elmanama AA, Laham NAA, Tayh GA. Antimicrobial susceptibility of bacterial isolates from burn units in Gaza. Burns. 2013;39(8):1612–8. 23664775 10.1016/j.burns.2013.04.011

[44] Rashid Mahmood A, Mansour HN. Study of Antibiotic Resistant Genes in Pseudomonas aeroginosa Isolated from Burns and Wounds. Arch Razi Inst. 2022;77(1):379–87. 10.22092/ARI.2021.356681.1893 PMC9288643 35891744

[45] Sami Awayid H, Qassim MS. Prevalence and Antibiotic Resistance Pattern of Methicillin-Resistant Staphylococcus aureus Isolated from Iraqi Hospitals. Arch Razi Inst. 2022;77(3):1147–56. 36618304 10.22092/ARI.2022.357391.2031 PMC9759256

[46] Khemiri M, Akrout Alhusain A, Abbassi MS, El Ghaieb H, Santos Costa S, Belas A, et al. Clonal spread of methicillin-resistant Staphylococcus aureus-t6065-CC5-SCCmecV-agrII in a Libyan hospital. J Glob Antimicrob Resist. 2017;1(10):101–5. 10.1016/j.jgar.2017.04.014 28729209

[47] Ali FA. Association Between Biofilm Formation Gene Bla exoU and Metallo and Extend Spectrum Beta-lactamase Production of Multidrug Resistance Pseudomonas aeruginosa in Clinical Samples. Comb Chem High Throughput Screen. 2021;25(7):1207–18. 10.2174/1386207324666210419112210 33874869

[48] Älgå A, Wong S, Shoaib M, Lundgren K, Giske CG, von Schreeb J, et al. Infection with high proportion of multidrug-resistant bacteria in conflict-related injuries is associated with poor outcomes and excess resource consumption: A cohort study of Syrian patients treated in Jordan. BMC Infect Dis. 2018;18(1):233. 29788910 10.1186/s12879-018-3149-y PMC5964734

[49] Joint Formulary Committee. Ceftriaxone - indications and dose. In: Joint formulary Committee. British National Formulary [BNF Online]. London: BMJ Group and the Royal Pharmaceutical Society; 2024.

[50] Fily F, Ronat JB, Malou N, et al. Post-traumatic osteomyelitis in Middle East war-wounded civilians: Resistance to first-line antibiotics in selected bacteria over the decade 2006–2016. BMC Infect Dis. 2021;19(1):257. 10.1186/s12879-019-3741-9. 10.1186/s12879-019-3741-9 PMC6357381 30704410

[51] Almehdar H, Yousef N, van den Boogaard W, Haider A, Kanapathipillai R, Al-Hodiani E, Zelikova E, Moh'd WG, Michel J, Malaeb R. Antibiotic susceptibility patterns at the Médecins Sans Frontières (MSF) Acute Trauma Hospital in Aden, Yemen: a retrospective study from January 2018 to June 2021. JAC Antimicrob Resist. 2024;6(2):dlae024. 10.1093/jacamr/dlae024. 10.1093/jacamr/dlae024 PMC10914454 38449518

[52] Murphy RA, Ronat JB, Fakhri RM, Herard P, Blackwell N, Abgrall S, et al. Multidrug-resistant chronic osteomyelitis complicating war injury in Iraqi civilians. Journal of Trauma - Injury, Infection and Critical Care. 2011;71(1):252–4. 10.1097/TA.0b013e31821b8622 21818032

[53] Chamoun K, Farah M, Araj G, Daoud Z, Moghnieh R, Salameh P, et al. Surveillance of antimicrobial resistance in Lebanese hospitals: Retrospective nationwide compiled data. Int J Infect Dis. 2016;1(46):64–70. 10.1016/j.ijid.2016.03.010 26996458

[54] O’Shea MK. Acinetobacter in modern warfare. Int J Antimicrob Agents. 2012;39:363–75. 22459899 10.1016/j.ijantimicag.2012.01.018

[55] Haraoui L, Sparrow A, Sullivan R, Burci G, Dewachi O, Abu-Sittah G et al. Armed conflicts and antimicrobial resistance: A deadly convergence. AMR Control 2019-2020. 2019 [cited 2025 Jan 31]: 69-73. Available at: http://resistancecontrol.info/wp-content/uploads/2019/05/Haraoui.pdf.

[56] Hujer KM, Hujer AM, Hulten EA, Bajaksouzian S, Adams JM, Donskey CJ, et al. Analysis of antibiotic resistance genes in multidrug-resistant Acinetobacter sp. isolates from military and civilian patients treated at the Walter Reed Army Medical Center. Antimicrob Agents Chemother. 2006;50(12):4114–23. 17000742 10.1128/AAC.00778-06 PMC1694013

[57] Towner KJ. Acinetobacter: an old friend, but a new enemy. Journal of Hospital Infection. 2009;73:355–63. 19700220 10.1016/j.jhin.2009.03.032

[58] Peleg AY, Seifert H, Paterson DL. Acinetobacter baumannii: Emergence of a successful pathogen. Clin Microbiol Rev. 2008;21:538–82. 18625687 10.1128/CMR.00058-07 PMC2493088

[59] United Nations News. Iraq must seize ‘brief window of opportunity’ to turn tide of instability**.** 2023. Accessed 22 May 2024. Available from: https://news.un.org/en/story/2023/02/1133097.

[60] Granata G, Petersen E, Capone A, Donati D, Andriolo B, Gross M, et al. The impact of armed conflict on the development and global spread of antibiotic resistance: a systematic review. Clin Microbiol Infect. 2024;30(7):858–65. 10.1016/j.cmi.2024.03.029. 38556213 10.1016/j.cmi.2024.03.029

[61] Mohamed AH, Mohamud MFY, Mohamud HA. Epidemiology and antimicrobial susceptibility pattern of uropathogens in patients with the community-and hospital-acquired urinary tract infections at a tertiary hospital in somalia. Jundishapur J Microbiol. 2020;13(9):1–7.

[62] Ragueh AA, Aboubaker MH, Mohamed SI, Rolain JM, Diene SM. Emergence of Carbapenem-Resistant Gram-Negative Isolates in Hospital Settings in Djibouti. Antibiotics (Basel). 2023;12(7):1132. 10.3390/antibiotics12071132. 37508230 10.3390/antibiotics12071132 PMC10376901

[63] Khogali A, Homeida A. Impact of the 2023 armed conflict on Sudan’s healthcare system. Public Health Challenges. 2023;2(4):e134. Available here: 10.1002/puh2.134. 10.1002/puh2.134 PMC12039653 40496780

[64] Salmiya MA. Stop the Gaza genocide immediately. The Lancet. 2024; Available from: https://linkinghub.elsevier.com/retrieve/pii/S0140673624001351. 10.1016/S0140-6736(24)00135-1 38761810

[65] Human Rights Watch. World Report 2023: Yemen Events of 2023. 2024. Accessed 22 May 2024. Available from: https://www.hrw.org/world-report/2023/country-chapters/yemen.

[66] Palattiyil G, Sidhva D, Seraphia Derr A, Macgowan M. Global trends in forced migration: Policy, practice and research imperatives for social work. Int Soc Work. 2022;65(6):1111–29.

[67] Guha-Sapir D, Scales SE. Challenges in public health and epidemiology research in humanitarian settings: experiences from the field. BMC Public Health. 2020;20(1):1761. 10.1186/s12889-020-09851-7. 33228599 10.1186/s12889-020-09851-7 PMC7684900

