# Supplementary material for: Conflict-associated wounds and burns infected with GLASS pathogens in the Eastern Mediterranean Region: A systematic review
Source: BMC Infect Dis. 2025 Feb 7;25:187. doi: 10.1186/s12879-025-10569-3 (PMC11806698; doi:10.1186/s12879-025-10569-3)
Supplement: Supplementary file 1 — Supplementary Material 1. [file 12879_2025_10569_MOESM1_ESM.docx]

**Additional File 1.1: Search Strategy**

**Academic Database Search**

A search strategy was generated and duplicated across all database searches. Modifications were made only where MESH terms differed. Databases searched using OVID search engine were as follows: EMBASE, Global Health, MEDLINE. In addition, databases searched individually were: Scopus and Index Medicus for the Eastern Mediterranean Region.

See Table 1 for an example of the search strategy used for EMBASE.

**Table 1: EMBASE search strategy**

| Search Strategy | EMBASE  January 2010 to May 2024 (date of search: 1 June 2024) |
| --- | --- |
| 1 | exp drug resistance/ |
| 2 | exp antibiotic resistance/ |
| 3 | exp multidrug resistance/ |
| 4 | exp microbial sensitivity test/ |
| 5 | exp carbapenem resistance/ or exp carbapenem resistant acinetobacter baumannii/ or exp carbapenem-resistant enterobacteriaceae/ or exp carbapenem resistant escherichia coli/ |
| 6 | exp carbapenem resistance/ or exp carbapenem resistant acinetobacter baumannii/ or exp carbapenem-resistant enterobacteriaceae/ or exp carbapenem resistant escherichia coli/ or exp carbapenem resistant klebsiella pneumoniae/ or exp carbapenem resistant pseudomonas aeruginosa/ |
| 7 | exp antibiotic resistance/ or exp antibiotic sensitivity/ |
| 8 | exp carbapenem resistance/ |
| 9 | (((antimicrobial* or antibiotic* or antifungal* or antiviral* or anti microbial* or anti biotic* or anti fungal* or anti viral* or drug or drugs or multidrug* or microbial) and (resist* or sensitiv* or susceptib*)) or AMR or AMS or Carbapenem resistan* or Trauma Infectious Disease Outcomes Stud*).mp. |
| 10 | 1 or 2 or 3 or 4 or 5 or 6 or 7 or 8 or 9 |
| 11 | exp wound assessment/ or exp missile wound/ or exp wound tissue/ or exp stab wound/ or exp wound complication/ or exp wound infection/ or exp surgical wound/ or exp wound/ or exp burn/ |
| 12 | *injury/ |
| 13 | injury/ |
| 14 | (wound* or injuries or injury or injure or injured or trauma*) |
| 15 | 11 or 12 or 13 or 14 |
| 16 | middle east/ or iran/ or iraq/ or lebanon/ or palestine/ or syrian arab republic/ or yemen/ |
| 17 | "africa south of the sahara"/ or sudan/ |
| 18 | north africa/ or libyan arab jamahiriya/ |
| 19 | exp Somalia/ |
| 20 | Afghanistan/ |
| 21 | (Afghan* or Iraq* or Somalia or Sudan* or Syria* or West Bank or Gaza or Yemen or Libya* or Djibouti or Palestin* or Lebanon* or Turk* or Jordan*). |
| 22 | 16 or 17 or 18 or 19 or 20 or 21 |
| 23 | exp warfare/ |
| 24 | exp conflict/ |
| 25 | war/ |
| 26 | (civilian* or refugee* or migrant* or immigrant* or emigrant* or displaced or conflict or combat or casualt* or victim*or enduring freedom or explosive device* or landmine* or land mine* or munition* or postconflict or post conflict or postwar or shrapnel or war or wars or warfare or warring or Arab spring).mp. |
| 27 | 23 or 24 or 25 or 26 |
| 28 | 10 and 15 and 22 and 27 |
| 29 | limit 28 to yr="2010 -Current" |

**Grey Literature Search:**

A grey literature search included relevant search terms being selected and searched individually in: Google Scholar (www.scholar.google.com), International Committee of Red Cross website (www.icrc.org) and Medicines Sans Frontieres website ([www.msf.org.uk](http://www.msf.org.uk)). Search terms included: “Afghanistan”, “Sudan”, “Iraq”, “Somalia”, Syria, “Gaza”, “Yemen”, “Libya”, “conflict”, “war”, “combat”, “wound”, “burns”, “civilian”.

**Additional File 1.2: Definitions**

| **Term** | **Abbreviation** | **Definition** |
| --- | --- | --- |
| Anti-microbial Resistance | AMR | An organism which has shown resistant, intermediate or non-susceptible results when tested on specified antimicrobials, according to standardised criteria.^15^ |
| Conflict-affected country in WHO EMRO |  | Countries that have been included in the World Bank’s List of Fragile and Conflict Situations from January 2010 – January 2024.^2^  As of February 2024, these include: Afghanistan, Iraq, Somalia, Sudan, Syrian Arab Republic, Yemen, Republic of, Libya, occupied Palestinian territory (including West Bank and Gaza), Djibouti, Lebanon. |
| Conflict-affected wound or burn |  | Any wound or burn obtained as a result of armed-conflict situations. Mechanisms of injuries may include: traumatic and non-accidental injury, gunshots, explosions (including burns, damage from unstable infrastructure), shrapnel injuries, injuries obtained during forced migration. |
| Global Antimicrobial Surveillance System Priority Pathogens^17^ | GLASS pathogens | Pathogens listed by the World Health Organisation to be prioritised in research and development.  These include:   - Carbapenem-resistant *Acinetobacter baumannii (A. baumannii).* - *Carbapenem-resistant Pseudomonas aeruginosa (P. aeruginosa).* - *Carbapenem-resistant and third generation cephalosporin resistant Enterobacteriaceae* (including *Escherichia coli (E. coli) and* *Klebsiella pneumoniae (K. pneumoniae*)). - Methicillin resistant *Staphylococcus aureus (S. aureus)*. |
| Multi-drug resistant organism | MDRO | Defined as an organism that is non-susceptible to 1 or more agents in 3 or more antimicrobial classes.^15^ |
| World Health Organisation Eastern Mediterranean Region | EMRO region | Region defined by the World Health Organisation, including 21 member states and the occupied Palestinian territory.^3^ |

**Selected GLASS Pathogens and Antimicrobials**

|  | Pathogen | Antimicrobial Class | Specific Antimicrobials |
| --- | --- | --- | --- |
| Gram Positive | Staphylococcus aureus | Glycopeptides  Penicillins | Vancomycin Methicillin |
| Gram Negative | Enterobacteriacae (*K. pneumoniae*, *E. coli*) | Carbapenems Third generation cephalosporins Fluoroquinolones  Aminoglycosides | Imipenem, Meropenem Cefotaxime, Ceftazidime, Ceftriaxone Ciprofloxacin, Levofloxacin  Gentamicin, Amikacin |
|  | Acinetobacter baumannii | Carbapenems Aminoglycosides | Ciprofloxacin, Levofloxacin  Gentamicin, Amikacin |
|  | Pseduomonas aeruginosa | Carbapenems Aminoglycosides | Ciprofloxacin, Levofloxacin  Gentamicin, Amikacin |

**Additional File 1.3: Quality Assessment of Included Studies**

The Joanna Briggs Institute checklists were used to assess study quality for all studies. Each domain was assessed and graded as “yes”, “no” or “unclear/ cannot tell”, which were allocated points 3, 0, and 1 respectively. An overall score was calculated and risk of bias was categorised according to: >21= low risk, 15-21= moderate risk, <15= high risk.

**Table 1: Quality assessment of studies included in this study according to Joanna Briggs checklist domains.**

| Study | Were the criteria for inclusion in the sample clearly defined? | Were the criteria for inclusion in the sample clearly defined? | Was the exposure measured in a valid and reliable way? | Were objective, standard criteria used for measurement of the condition? | Were confounding factors identified? | Were strategies to deal with confounding factors stated? | Were the outcomes measured in a valid and reliable way? | Was appropriate statistical analysis used? | Overall Score | Risk of Bias Category |
| --- | --- | --- | --- | --- | --- | --- | --- | --- | --- | --- |
| Dau et al., 2011 | N | Y | Y | Y | Y | N | Y | N | 15 | Moderate |
| Elmanama et al., 2013 | Y | U | Y | Y | Y | Y | Y | Y | 22 | Low |
| Teicher et al., 2014 | U | Y | Y | Y | Y | N | U | N | 14 | High |
| Rafei et al., 2015 | N | N | U | N | U | Y | Y | Y | 11 | High |
| Zorgani et al., 2015 | Y | U | Y | Y | N | N/A | U | Y | 14 | High |
| Khemiri et al., 2017 | N | Y | U | N/A | N | N/A | Y | N | 8 | High |
| Aljanaby et al., 2017 | N | N | Y | Y | N | N/A | Y | N | 9 | High |
| Nasser et al., 2018 | N | Y | Y | Y | N | N/A | Y | N/A | 12 | Moderate |
| Alga et al., 2018 | Y | Y | Y | Y | Y | N | Y | N | 18 | Low |
| Bourgi et al., 2020 | Y | Y | N | N | Y | Y | U | Y | 16 | Moderate |
| Hateet et al., 2021 | N | N | N/A | U | N | U | Y | U | 7 | High |
| M'Aiber et al., 2022 | Y | Y | Y | Y | Y | Y | Y | Y | 24 | Low |
| Yaacoub et al., 2022 | Y | Y | Y | N/A | Y | Y | Y | Y | 22 | Low |
| Awayid et al., 2022 | N | N | Y | Y | N | N/A | Y | U | 10 | High |
| Mahmood et al., 2022 | N | N | N | Y | N | N/A | Y | N | 9 | High |
| Ali et al., 2022 | N | N | N | Y | Y | Y | Y | Y | 15 | Moderate |
| Al Miyah et al., 2023 | Y | Y | Y | Y | N | N/A | Y | Y | 18 | Moderate |
| Ali et al., 2024 | Y | U | Y | U | Y | Y | Y | Y | 20 | Moderate |
| Khalid et al., 2024 | Y | U | U | Y | Y | N | Y | N | 14 | High |
