# Supplementary material for: Conflict-associated wounds and burns infected with GLASS pathogens in the Eastern Mediterranean Region: A systematic review
Source: BMC Infect Dis. 2025 Feb 7;25:187. doi: 10.1186/s12879-025-10569-3 (PMC11806698; doi:10.1186/s12879-025-10569-3)
Supplement: Supplementary file 2 — Supplementary Material 2. [file 12879_2025_10569_MOESM2_ESM.docx]

**Additional File 2.1: Study Sample and Patient Numbers**

**Table 2**: A table showing the number of patient participants and isolates identified in each study, grouped according to country.

*SA: S. aureus, KP: K. pneumoniae, EC: E. coli, PA: P. aeruginosa, AB: A. baumannii*

| **Country** | **Author** | **Patients included (tested)** | **Patients from which positive isolates found** | **Total number samples taken** | **Total number positive isolates** | **Wound Isolate numbers (positive)** | **Gram positive number** | **Gram negative number** | **SA** | **KP** | **EC** | **Enterobacteriaceae** | **AB** | **PA** | **Total number of Isolates of interest** |
| --- | --- | --- | --- | --- | --- | --- | --- | --- | --- | --- | --- | --- | --- | --- | --- |
| Lebanon | **Yaacoub et al. 2022** | 672 | - | 3204 | 1149 | 1149 | 182 | 152 | 171 | 18 | 25 | - | 7 | 46 | 267 |
|  | **Rafei et al. 2015** |  | 116 |  | 116 | 68 | - | 68 | - | - | - | - | 68 | - | 68 |
|  | **Bourgi et al. 2020** | 475 | 261 |  | 277 | 154 | 58 | 97 | 58 | 9 | 10 | - | 22 | 45 | 144 |
|  | **TOTAL** |  |  |  |  | 1371 | 240 | 317 | 229 | 27 | 35 | 0 | 97 | 91 | 479 |
| Iraq | **M'aiber et al. 2022** | 174 | 325 | 421 | 409 | 349 | 217 | 192 | 197 | 22 | 30 | - | 3 | - | 252 |
|  | **Khalid et al. 2024** |  |  |  | 117 | 117 | - | 117 | - | - | - | - | 117 | - | 117 |
|  | **al Miyah et al. 2022** | 450 |  | 450 | 87 | 87 | - | 87 | - | 87 | - | - | - | - | 87 |
|  | **Hateet et al. 2021** | 105 |  | 105 | 105 | 105 | 23 | 83 | 18 | 7 | 8 | - | - | 21 | 54 |
|  | **Awayid et al. 2022** |  |  | 150 | 64 | 64 | 64 |  | 64 | - | - | - | - | - | 64 |
|  | **Mahmood et al. 2022** |  |  | 200 | 31 | 31 | - | 31 | - | - | - | - | - | 31 | 31 |
|  | **Ali et al. 2024** | 150 | 62 | 150 | 80 | 80 | 48 | 32 | 48 | - | - | - | - | 32 | 80 |
|  | **Ali et al. 2022** |  |  | 227 | 40 | 23 | - | 23 | - | - | - | - | - | 23 | 23 |
|  | **Aljanaby et al. 2017** |  |  | 144 | 43 | 19 | - | 19 | - | 19 | - | - | - | - | 19 |
|  | **TOTAL** |  |  |  |  | 875 | 352 | 584 | 327 | 135 | 38 | 0 | 120 | 107 | 727 |
| Libya | **Zorgani et al. 2015** | 560 (across 2009 and 210) | - | 78 | 78 | - | 78 | - | - | - | - | - | - | - | 78 |
|  | **Khemiri et al. 2017** |  | - | 32 | 32 | 32 | 32 |  | 32 | - | - | - | - | - | 32 |
|  | **Dau et al. 2011** | 1200 | 498 | 1200 | 764 | 764 | 178 | 586 |  | 86 | 107 |  | 144 | 92 | 429 |
|  | **TOTAL** |  |  |  |  | 874 | 288 | 586 | 110 | 86 | 107 | 0 | 144 | 92 | 539 |
| Gaza | **Elmanama et al. 2013** | 118 | 53 | 118 | 53 | 53 | 5 | 48 | 5 | 1 | 3 | 20 | 1 | 27 | 57 |
|  | **TOTAL** |  |  |  |  |  | 5 | 48 | 5 | 1 | 3 | 20 | 1 | 27 | 57 |
| Yemen | **Nasser et al. 2018** | 99 | 46 | 99 | 46 | 46 |  | 46 | - | - | - | - | - | 46 | 46 |
|  | **Total** |  |  |  |  |  | 0 | 46 | 0 | 0 | 0 | 0 | 0 | 46 | 46 |
| Jordan | **Alga et al. 2018** | 457 | 49 | 81 | 49 | 49 | 15 | 34 | 15 | 11 | 8 | - | 5 | 12 | 51 |
|  | **Teicher et al. 2014** | 345 | 61 |  | 67 | 67 | 24 | 19 | 19 |  | 8 | - | 6 | 10 | 43 |
|  | **Total** |  |  |  |  | 116 | 39 | 53 | 34 | 11 | 16 | - | 11 | 22 | 94 |

**Additional File 2.3: Multi-drug Resistant Isolate Results and Definitions**

**Table 2:** Table showing the multidrug resistance values for Gram negative GLASS pathogens identified. Ordered according to date of publication. *XDR: extensively drug resistant. MDRO: Multi-drug resistant organism.*

| **Study** | **Year** | **Country** | **MDRO: *E. coli*** | **MDRO: *K. pneumoniae*** | **MDRO: *P. aeruginosa*** | **MDRO: *A. baumannii*** | **MDRO definition** |
| --- | --- | --- | --- | --- | --- | --- | --- |
| **Elmanama et al.** | 2013 | Gaza | - | - | 100% | - | “Resistant to most of the antimicrobials used” |
| **Aljanaby et al.** | 2017 | Iraq | - | 42.1% (47.36% XDR) | - | - | Resistant to a minimum of at least 3 different classes of antibiotics. |
| **Nasser et al.** | 2018 | Yemen | - | - | 65.20% | - | Non- susceptible to at least one agent in three or more antimicrobial categories. |
| **Alga et al.** | 2018 | Jordan | 100% | 82% | 17% | 100% | Resistance to at least one antibiotic from three or more relevant antibiotic groups |
| **M’aiber et al.** | 2022 | Iraq | 96.70% | 90.90% | - | - | No sensitivity to three or more antibiotic classes. |
| **Al Miyah et al.** | 2022 | Iraq | - | 63% | - | - | Resistant to more than 3 drugs. |
| **Mahmood et al.** | 2022 | Iraq | - | - | 9.68% | - | No definition specified |
| **Yaacoub et al.** | 2022 | Lebanon | 88% | 83% | 7.60% | 1.60% | Non-susceptibility to at least one agents in three or more antimicrobial categories. |
| **Bourgi et al.** | 2023 | Lebanon | - | - | Majority | Majority | Acquired resistant to one or more agent in at least three antimicrobial categories. |
